# A Metabolomics Approach for Predicting OATP1B-Type Transporter-Mediated Drug–Drug Interaction Liabilities

**DOI:** 10.3390/pharmaceutics14091933

**Published:** 2022-09-13

**Authors:** Yang Li, Yan Jin, Hanieh Taheri, Keith T. Schmidt, Alice A. Gibson, Stefan A. J. Buck, Eric D. Eisenmann, Ron H. J. Mathijssen, William D. Figg, Sharyn D. Baker, Alex Sparreboom, Shuiying Hu

**Affiliations:** 1 Division of Pharmaceutics and Pharmacology, College of Pharmacy & Comprehensive Cancer Center, The Ohio State University, Columbus, OH 43210, USA; 2 Division of Outcomes and Translational Sciences, College of Pharmacy & Comprehensive Cancer Center, The Ohio State University, Columbus, OH 43210, USA; 3 Clinical Pharmacology Program, Office of the Clinical Director, National Cancer Institute, Bethesda, ML 20892, USA; 4 Department of Medical Oncology, Erasmus Medical Center Cancer Institute, Dr. Molewaterplein 40, 3015 GD Rotterdam, The Netherlands

**Keywords:** endogenous biomarkers, OATP1B, taxane, drug–drug interactions

## Abstract

In recent years, various endogenous compounds have been proposed as putative biomarkers for the hepatic uptake transporters OATP1B1 and OATP1B3 that have the potential to predict transporter-mediated drug–drug interactions (DDIs). However, these compounds have often been identified from top–down strategies and have not been fully utilized as a substitute for traditional DDI studies. In an attempt to eliminate observer bias in biomarker selection, we applied a bottom–up, untargeted metabolomics screening approach in mice and found that plasma levels of the conjugated bile acid chenodeoxycholate-24-glucuronide (CDCA-24G) are particularly sensitive to deletion of the orthologous murine transporter Oatp1b2 (31-fold increase vs. wild type) or the entire Oatp1a/1b(−/−)cluster (83-fold increased), whereas the humanized transgenic overexpression of hepatic OATP1B1 or OATP1B3 resulted in the partial restoration of transport function. Validation studies with the OATP1B1/OATP1B3 inhibitors rifampin and paclitaxel in vitro as well as in mice and human subjects confirmed that CDCA-24G is a sensitive and rapid response biomarker to dose-dependent transporter inhibition. Collectively, our study confirmed the ability of CDCA-24G to serve as a sensitive and selective endogenous biomarker of OATP1B-type transport function and suggests a template for the future development of biomarkers for other clinically important xenobiotic transporters.

## 1. Introduction

Membrane transporters, including the ATP-binding cassette (ABC) and solute carrier (SLC) superfamilies, are increasingly recognized to play important roles in the absorption, distribution, metabolism, and excretion of many structurally and therapeutically diverse classes of drugs [1]. Therefore, the continuing identification of transporter substrates and/or inhibitors provides key tools to increase our understanding of the clinical pharmacokinetic properties of xenobiotics as victims or perpetrators of pharmacokinetic drug–drug interactions (DDIs) [2]. In recent years, there has been an increasing awareness that substrates of classic drug transporters also include various endogenous metabolites, and significant advances have been made in evaluating such compounds as potential clinical biomarkers to predict transporter-mediated DDIs [3]. In particular, metabolomics, a systems-wide analysis of metabolites in biological samples, has been applied as a strategy to identify endogenous substrates of drug transporters that can serve as biomarkers in both humans and other mammalian species [4]. In addition, the availability of engineered animal models that are deficient in a particular transporter or that carry the transgenic overexpression of human transporters has facilitated the discovery of endogenous substrates of drug transporters [5,6]. With appropriate in vitro and in vivo validation strategies, such animal models could be employed to predict transporter-mediated DDI liabilities in the context of commonly employed polypharmacy approaches to therapeutics.

**Figure 1:**
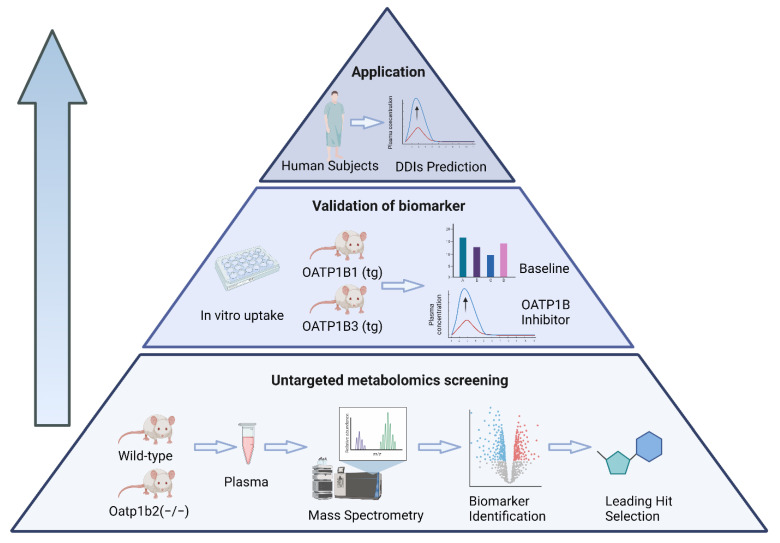
Schematic depiction of a bottom–up, untargeted metabolomics screening approach for predicting OATP1B-type transporter-mediated DDI liabilities.

The SLC class of organic anion transporting polypeptides (OATPs) is of particular interest in connection with DDI predictions, as these uptake transporters are highly expressed in various organs of elimination of relevance to a rapidly increasing number of clinically important drugs [7]. In humans, the family members OATP1B1 and OATP1B3 (collectively referred to throughout as ‘OATP1B’) are expressed on the sinusoidal membrane of hepatocytes, and these proteins play key roles, either individually or collectively, in the hepatic uptake of many drugs [8,9,10]. In scenarios where this process is rate-limiting, the inhibition of OATP1B can lead to defective elimination, result in sudden increases in the plasma concentration for drugs that are substrates of these transporters, and ultimately increase the risk of therapy-related side effects [11]. Based on these considerations, both the US Food and Drug Administration and the European Medicines Agency stipulate, under certain conditions, that sponsors perform studies to evaluate whether new investigational small molecule drugs have intrinsic OATP1B-inhibitory properties. Although endogenous substrates of OATP1B have been previously proposed as biomarkers to predict OATP1B-mediated DDIs [3,4,12,13], further validation studies are warrant to conclusively demonstrate their utility as a substitute for traditional DDI studies. One potential reason underlying this limitation resides in the fact that the specificity and sensitivity of most proposed biomarkers have not been directly demonstrated. In an attempt to eliminate observer bias in biomarker selection, we describe here the results of a bottom–up, untargeted metabolomics screening approach for OATP1B biomarker discovery in various transporter-deficient and humanized transgenic mouse models and validated lead hits in mice and human subjects receiving OATP1B modulators of varying inhibitory potency (Figure 1).

## 2. Materials and Methods

### 2.1. Animals

For all experiments, age- and sex-matched mice (8–12 weeks old) were used. All animals were housed in a temperature-controlled environment with a 12 h light cycle, given a standard chow diet and water ad libitum, and were housed and handled according to and approved by the University Laboratory Animal Resources Animal Care and Use Committee at The Ohio State University, under an approved protocol (2015A00000101-R2). Wild-type mice on a DBA/1LacJ or FVB background were purchased from The Jackson Laboratory (Bar Harbor, ME, USA) or Taconic Biosciences (Rensselaer, NY, USA). Mice with a deficiency of Oatp1b2 (Oatp1b2(−/−) mice), the single murine orthologue of OATP1B1 and OATP1B3 in humans, on a DBA/1LacJ background were kindly provided by Drs. Richard B. Kim (Western University, London, Ontario, Canada) and Jeffrey L. Stock (Pfizer, Groton, CT, USA). Murine Oatp1a/1b cluster-knockout mice (Oatp1a/1b(−/−) mice) and Oatp1a/1b(−/−) mice with transgenic hepatic expression of OATP1B1 (OATP1B1(tg) mice) or OATP1B3 (OATP1B3(tg) mice) were kindly provided by Dr. Alfred H. Schinkel (The Netherlands Cancer Institute, Amsterdam, The Netherlands).

### 2.2. Untargeted Metabolomics

Plasma samples from untreated wild-type mice and Oatp1b2(−/−) mice were analyzed for structurally named and unknown molecules using a non-targeted semi-quantitative liquid chromatography-tandem mass spectrometry (LC-MS/MS) and gas chromatography-mass spectrometry platforms (Metabolon, Durham, NC, USA). Compounds were identified by comparison with library entries of purified standards or recurrent unknown entities. This library is based on authenticated standards and contains the retention time/index (RI), mass to charge ratio (*m*/*z*), and chromatographic data (including MS/MS spectral data) on all molecules present. Peaks were quantified using area under the curve (AUC), where each biochemical in raw area counts is rescaled to set the median equal to 1 and missing values were imputed. Welch’s two-sample *t*-test was used to identify biochemicals that differed significantly between experimental groups.

### 2.3. Murine Pharmacokinetic Studies

Pharmacokinetic studies were performed following an established protocol described in detail elsewhere [14]. The influence of the OATP1B inhibitors rifampin (20 mg/kg, i.v.; formulated in 10% DMSO and 90% saline), paclitaxel (10 mg/kg, i.v.; formulated in 12.5% Cremophor EL, 12.5% ethanol and 75% saline), or the Cremophor EL vehicle alone on the plasma concentrations of chenodeoxycholate-24-glucuronide (CDCA-24G), the lead hit identified from the metabolomics analysis, was performed in wild-type mice and Oatp1b2(−/−) mice. In these experiments, serial whole blood samples were collected in heparinized tubes immediately prior to drug administration (pre-dose) and at 0.25, 0.5, 1, 2, and 4 h after administration from the submandibular vein for the initial three time-points, from the retro-orbital sinus vein for the subsequent two time-points, and by cardiac puncture at the terminal time-point. Whole blood samples were centrifuged at 11,000 rpm for 5 min; then, the plasma supernatants were collected and stored at −80 °C until analysis by a validated method based on LC-MS/MS.

### 2.4. In Vitro Studies

The generation of human embryonic kidney (HEK293) cells (ATCC) engineered to overexpress OATP1B1, Oatp1b2, or empty vector, the cell culture conditions, and general details of uptake studies have been reported in detail elsewhere [15]. Briefly, cells were grown to 90% confluence on poly-lysine coated multi-well plates. For uptake, cells were briefly rinsed with warm 1× PBS and incubated for 15 min in the presence or absence of vehicle or the OATP1B inhibitors paclitaxel (10 μM), prepared in serum-free and phenol-red free DMEM. The pre-treatment medium was rinsed off with warm 1× PBS followed by the addition of the test compounds for an incubation period of 5 min. After incubation, transport was halted by aspirating the medium and rinsing the cells three times with ice-cold 1× PBS. After lysing cells with 0.1 N HCl, and subsequent adding methanol, 20 μL aliquots were mixed with 60 μL of acetonitrile containing relevant internal standards. After vortex mixing, the mixtures were centrifuged, and the supernatants were used for quantitative analysis by LC-MS/MS. Drug uptake results were normalized to total protein content and then to data obtained in cells carrying an empty vector plasmid, which was set to 100%.

### 2.5. Clinical Studies

Samples from patients with solid tumors were collected from 3 clinical studies conducted in accordance with the principles of the Declaration of Helsinki, and all study protocols were approved by Institutional Review Boards at either the National Cancer Institute (Bethesda, MD, USA) or the Erasmus Medical Center Cancer Institute (Rotterdam, The Netherlands). All patients provided written informed consent for their blood samples and medical information to be used for research purposes. Details of these studies, including trial design and inclusion and exclusion criteria, have been reported previously [16,17,18]. In the first study, patients received paclitaxel formulated in Cremophor EL-ethanol (Taxol; Bristol-Myers Squibb, New York, NY, USA) as a 1 h i.v. infusion at a dose of 50 or 100 mg/m^2^, and blood samples were collected before infusion and at 0.5, 0.92, 2.5, and 4 h after the start of infusion [18]. In the second study, patients were randomized to receive paclitaxel either formulated as a Cremophor EL-free, albumin bound nanoparticle (Abraxane, ABI-007; Celgene, Summit, NJ, USA) as a 30-min i.v. infusion at a dose of 260 mg/m^2^ or in Cremophor EL-ethanol (Taxol) as a 3 h i.v. infusion at a dose of 175 mg/m^2^. Blood samples were collected before the start of the infusion, 15 min (Abraxane) or 1.5 h (Taxol) after the start of the infusion, 2 to 5 min before the end of the infusion, and at 30 min and 1, 1.5, 2, 4, 6, 8, 12, 24, 48, and 72 h after the end of the infusion. Plasma concentrations of paclitaxel were measured using a validated method based on LC-MS/MS [17]. In the third study, patients received cabazitaxel (Jevtana; Sanofi, Bridgewater, NJ, USA) as a 1 h i.v. infusion at a dose of 12.5, 20, or 25 mg/m^2^. Blood samples were obtained pre-infusion and at 0.5, 0.92, 1.08, 1.25, 1.5, 2, 3, 5, 7, 11–13, 24, and 192 after the start of infusion. Plasma concentrations of cabazitaxel was measured using a validated LC-MS/MS method [16].

### 2.6. Quantification of Endogenous OATP1B Biomarkers in Plasma

Plasma concentrations of levels of CDCA-24G and glycochenodeoxycholate-3-sulfate (GCDCA-S) were determined using LC-MS/MS using a Vanquish UHPLC coupled with a Quantiva triple quadrupole mass spectrometer (Thermo Fisher Scientific, Waltham, MA, USA) [19]. An Accucore aQ column (50 × 2.1 mm, dp = 2.6 μm; Thermo Fisher Scientific) was protected by a C18 AQUASIL guard cartridge (2.1 mm × 10 mm, dp = 3 μm; Thermo Fisher Scientific). The injection volume of sample was 5.0 μL. The temperature of the autosampler rack was 4 °C, and the temperature of the column was maintained at 20 °C. The mobile phase was composed of solvent A (2 mmol ammonium acetate in water) and solvent B (100% methanol), and the total run time was 4.6 min. The gradient conditions were as follows: 0–0.5 min, 45% B; 0.5–2.0 min, 45 to 90%, 2.0–4.0 min, 90% B; 4.0–4.6 min, 45% B, delivered at a flow rate of 0.4 mL/min. The MS assay setting with the positive voltage applied to the ESI capillary was set at 3791 V, and the capillary temperatures was 335 °C with a vaporizer temperature of 350 °C. Argon was used as the collision gas at a pressure of 1.5 mTorr. Precursor molecular ions, and product ions were recorded for the confirmation and detection of GCDCA-S (528.4 > 448.3) and CDCA-24G (567.4 > 391.1), using [^2^H_5_]-GCDCA-S (535.5 > 453.3) and [^2^H_5_]-CDCA-24G (572.5 > 396.1) as respective internal standards. The lower limit of quantitation (LLOQ) for both analytes was determined to be 0.5 ng/mL. The within-run and between-run precisions for quality control samples run on several consecutive days were always within 13.8%, while the deviation from nominal values ranged from −8.15% to 10.1%.

Plasma concentrations of coproporphyrin I (CP-I) and III (CP-III) were determined similarly by LC-MS/MS. In brief, separation was accomplished using an Xbridge BEH Shield RP18 column (100 × 3.0 mm, dp = 2.5 μm; Waters, Milford, MA, USA), protected by an ACQUITY UPLC BEH Shield RP18 VanGuard column (2.1 mm × 5 mm, dp = 1.7 μm; Waters). The injection volume of the sample was 2.0 μL. The temperature of the autosampler rack was 4 °C, and the temperature of the column was maintained at 40 °C. The mobile phase was composed of solvent A (10 mmol ammonium acetate in water) and solvent B (0.1% formic acid in 100% acetonitrile), with a total run time of 7 min. The gradient conditions were as follows: 0–3.0 min, 40 to 60% B; 3.0–5.0 min, 60% B, 5.0–7.0 min, 40% B, delivered at a flow rate of 0.45 mL/min. The MS assay setting with the positive voltage applied to the ESI capillary was set at 3154 V, and the capillary temperatures was 380 °C with a vaporizer temperature of 400 °C. Argon was used as the collision gas at a pressure of 1.5 mTorr. Precursor molecular ions and product ions were recorded for confirmation and detection of CP-I (655.4 > 596.4) and CP-III (655.4 > 596.3), using [^2^H_4_]-CP-III (659.4 > 600.3) as the internal standard for both analytes. The LLOQ for both analytes were determined to be 1 ng/mL, and the within-run and between-run precisions as well as the accuracy were always within acceptable limits.

### 2.7. Pharmacokinetic Analysis

Pharmacokinetic parameters were derived from non-compartmental analysis using Phoenix WinNonlin version 8.0 (Certara, Princeton, NJ, USA). The peak plasma concentration (C_max_) was determined by visual inspection of the data from the concentration–time curves. The linear trapezoidal rule was used to obtain the area under the plasma concentration–time curve (AUC). The C_max_ to baseline ratio (C_max_R) instead of the reference method that relies on the AUC to control ratio (AUCR) was used to evaluate the modulation of OATP1B function, as samples from an untreated group were not available in our study, and previous studies [13,20] reported that such an approach provides data of equivalent utility. In the calculation of C_max_ ratio of the endogenous biomarkers, the control values were set as the baseline concentration of corresponding patient.

### 2.8. Statistical Analysis

All data are presented as mean ± SEM before and/or after normalization to baseline values and are expressed as a percentage, unless stated otherwise. All experiments were performed using multiple replicates and were performed independently on at least two separate occasions. An unpaired two-sided Student’s *t*-test with Welch’s correction was used for comparisons between two groups, and a one-way ANOVA with Dunnett’s post hoc test was used for comparing more than two groups. *p* < 0.05 was used as the cutoff for statistical significance across all analyses.

## 3. Results

### 3.1. Identification and Validation of OATP1B Biomarkers

**Figure 2:**
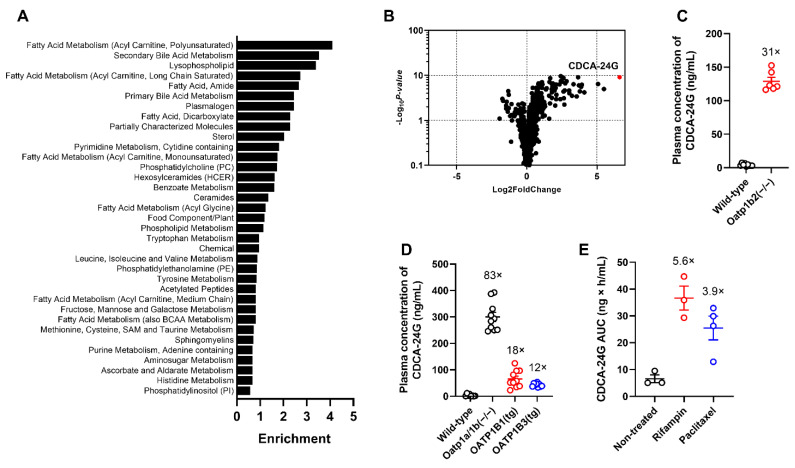
Identification of endogenous biomarkers of OATP1B-type transporters. (**A**) Enrichment of bile acid metabolic pathways, based on differential metabolite profiles, using plasma samples from untreated wild-type mice or Oatp1b2(−/−) mice. (**B**) Differentially circulating levels of endogenous metabolites in plasma of wild-type mice or Oatp1b2(−/−) mice. Positive fold change indicates higher plasma concentration in Oatp1b2(−/−) mice (*n* = 6 per group). (**C**) Validation of altered plasma levels of CDCA-24G in wild-type mice or Oatp1b2(−/−) mice (*n* = 6 per group). (**D**) Differential plasma levels of CDCA-24G in wild-type mice, Oatp1a/1b (−/−) mice, OATP1B1(tg) mice, and OATP1B3 (tg) mice (*n* = 6–10 per group). (**E**) Plasma AUC of CDCA-24G in wild-type mice receiving rifampin (20 mg/kg, i.v.) or paclitaxel (10 mg/kg, i.v.), (*n* = 3–4 per group). All data are presented as mean ± SEM.

To demonstrate the direct modulation of hepatic OATP1B function following the administration of OATP1B inhibitors, the identification of sensitive and selective biomarkers is required to guide the selection of optimal doses and schedules to be used in polypharmacy regimens and avoid potentially harmful DDIs. We hypothesized that this can be accomplished by probing for naturally occurring metabolites that are increased in plasma samples of untreated Oatp1b2(−/−) mice, the single murine homologue of human OATP1B1/3, by conducting untargeted metabolomic analyses using MS/MS-based platforms. A preliminary analysis of this metabolomics screen indicated that the observed metabolite profiles in the plasma of wild-type mice and Oatp1b2(−/−) mice were discriminative in a principal component analysis (Figure S1A). The differential metabolites among mouse genotypes were predominantly related to primary and secondary bile acid metabolism and fatty acid metabolism after pathway enrichment analysis (Figure 2A and Figure S1B). Consistent with previous reports indicating that certain primary and secondary bile acids are transported substrates of OATP1B-type transporters [21,22], we found that several of such bile acids had substantially increased circulating levels in Oatp1b2(−/−) mice (Figure 2B). Of the 919 compounds with detectable levels in mouse plasma, 198 (21.5%) showed higher plasma concentrations in Oatp1b2(−/−) mice (Table S1). The largest increase (≈98-fold vs. wild-type mice based on semi-quantitative analysis) was observed for CDCA-24G, which is a glucuronic acid conjugate of chenodeoxycholic acid that was previously reported to be a transported substrate of OATP1B1 and OATP1B3, and explored for clinical characterization [20,23]. For these reasons, CDCA-24G was selected for further validation studies in vitro and in vivo.

In vitro uptake studies in engineered cell-based models confirmed that CDCA-24G was efficiently transported by both human and murine OATP1B-type transporters and that this transport mechanism was sensitive to a known OATP1B inhibitor (Figure S2). Confirmation studies employing a validated, quantitative analytical method showed that at baseline, plasma levels of CDCA-24G were >30-fold higher in Oatp1b2(−/−) mice compared to wild-type mice (Figure 2C). We previously demonstrated that the rate of hepatic glucuronidation is unchanged in Oatp1b2(−/−) mice and that the reaction velocity of this process is negligible in intestinal microsomes regardless of mouse genotype [24]. This suggests that the altered systemic levels of CDCA-24G in Oatp1b2(−/−) mice cannot be explained by an intrinsically altered ability to metabolize bile acids via glucuronidation, and it supports instead a causal relationship with defective hepatic uptake transport.

One possible limitation of the Oatp1b2-deficient model used is the fact that, unlike in humans, mouse hepatocytes express multiple members of Oatp1a, which is a related subfamily of transporters that can potentially provide compensatory restoration of function when Oatp1b2 is lost [25]. Indeed, we found that baseline plasma levels of CDCA-24G in Oatp1a/1b(−/−) mice, which are deficient in all Oatp1a and Oatp1b isoforms, were >80 times higher than in wild-type mice, suggesting that Oatp1a partially contributes to the hepatobiliary processing of CDCA-24G. Notably, the introduction of human OATP1B1 or human OATP1B3 into livers of Oatp1a/1b(−/−) mice was associated with baseline levels of CDCA-24G that were 5- to 7-fold lower than values in Oatp1a/1b(−/−) mice (Figure 2D), thereby providing partial restoration of function. In comparison with wild-type mice, the observed changes in plasma levels of other commonly used markers of OATP1B function, including CP-I (Figure S3A) and CP-III (Figure S3B), in Oatp1a/1b(−/−) mice or in animals with transgenic expression of OATP1B1 or OATP1B3 were much more modest than those observed with CDCA-24G.

We next evaluated the influence of treatment with the known OATP1B inhibitors rifampin (20 mg/kg, i.v.) or paclitaxel (10 mg/kg, i.v.) on plasma concentrations of CDCA-24G in wild-type mice and found that the administration of these agents caused a transient, statistically significant increase in circulating levels (Figure 2E and Figure S3C,D). This suggests that CDCA-24G can serve as a sensitive and suitable endogenous biomarker to evaluate the effect of xenobiotics on OATP1B transporter function in vivo.

### 3.2. Implementation of CDCA-24G Analysis to Evaluate Modulation of OATP1B Function in Humans

**Figure 3:**
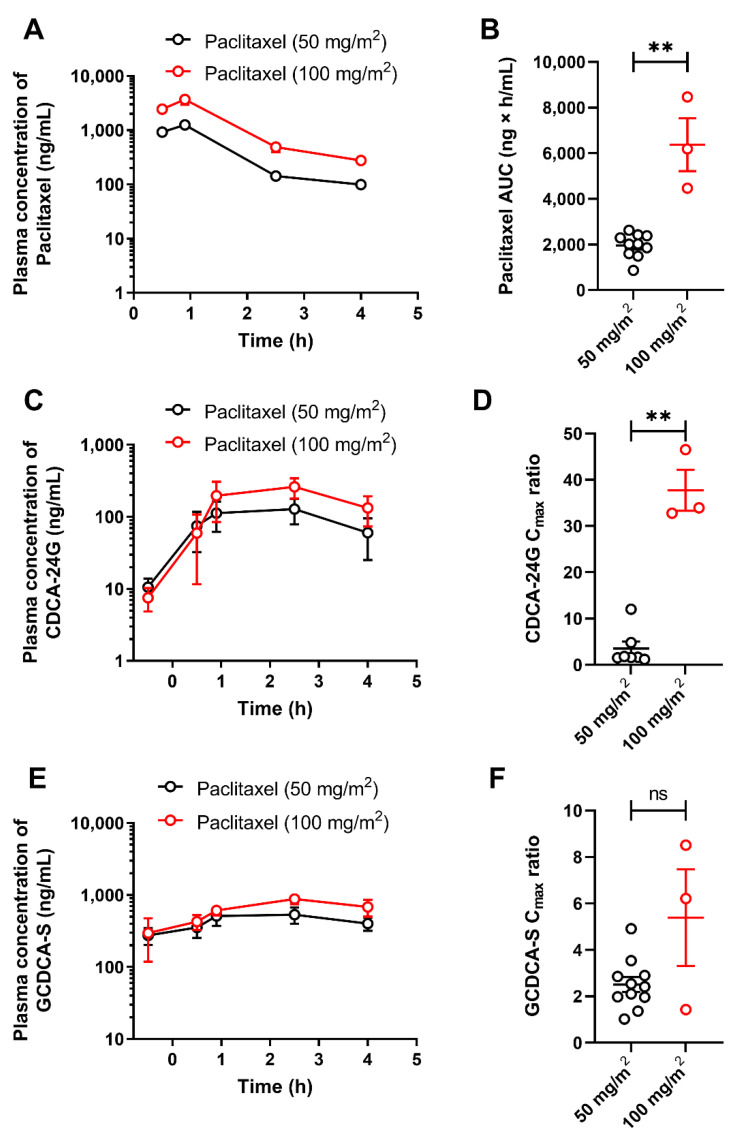
Influence of paclitaxel (Taxol) on OATP1B biomarkers. Plasma concentrations (**A**) and AUCs (**B**) of paclitaxel in patients after i.v. infusion of paclitaxel at a dose of 50 or 100 mg/m^2^. Plasma concentrations (**C**) and C_max_ to baseline ratio (**D**) of CDCA-24G, and plasma concentrations (**E**) and C_max_ to baseline ratio (**F**) of GCDCA-S in the same patients, ** *p* < 0.01, (*n* = 3–11). All data are presented a mean ± SEM.

To demonstrate the translational utility of CDCA-24G as a clinical biomarker of OATP1B function, several analyses were next performed on samples obtained from patients with cancer receiving treatment with paclitaxel (Taxol). The observed concentrations of paclitaxel after the administration of 50 or 100 mg/m^2^ doses were in the range predicted to have potential OATP1B-inhibitory effects (Figure 3A) [26], and the systemic exposure to paclitaxel itself was dependent on the dose administered (Figure 3B). In this cohort of patients, plasma levels of CDCA-24G were elevated, reached the peak concentration at 2.5 h after initiation of the paclitaxel infusion (Figure 3C), and the observed phenotypic changes occurred in a dose-dependent manner (Figure 3D). The levels of GCDCA-S, another bile acid previously proposed as an OATP1B substrate, were also modestly increased in manner dependent on the paclitaxel dose (Figure 3E), although the differences did not reach statistical significance (Figure 3F).

**Figure 4:**
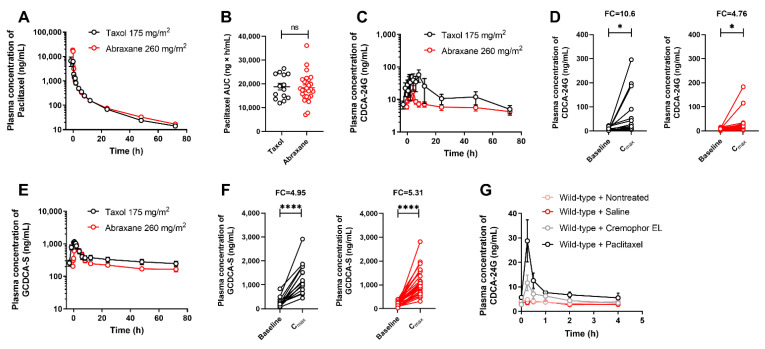
Influence of paclitaxel formulation on OATP1B biomarkers. (**A**) Plasma concentrations of paclitaxel and (**B**) AUC of paclitaxel in patients after i.v. infusion of Taxol (175 mg/m^2^) or Abraxane (260 mg/m^2^). (**C**) Plasma concentrations of CDCA-24G, (**D**) plasma concentration of CDCA-24G at baseline and C_max_ in patients receiving Taxol or Abraxane. (**E**) Plasma concentrations of GCDCA-S, (**F**) plasma concentration of GCDCA-S at baseline and C_max_ in patients receiving Taxol or Abraxane, * *p* < 0.05; **** *p* < 0.0001, (*n* = 13–26). (**G**) Plasma concentrations of CDCA-24G in untreated wild-type mice, or after receiving saline (5 mL/kg, i.v.), Cremophor EL/ethanol (1:1, *v*/*v*, 5 mL/kg, i.v.), or paclitaxel (Taxol; 10 mg/kg, 5 mL/kg, i.v.), (*n* = 3–5 per group). All data are presented as mean ± SEM.

Previous studies have suggested that Cremophor EL, a castor oil derivative used as a pharmaceutical excipient in some (e.g., Taxol) but not all (e.g., Abraxane) formulations of paclitaxel, may inhibit the function of OATP1B-type transporters both in vitro [27] and in vivo in mice [28]. To obtain evidence of this phenomenon in humans, we performed comparative studies in which patients received treatment with either Taxol or Abraxane. In these subjects, doses of Taxol and Abraxane were selected such that the plasma concentrations (Figure 4A) and overall systemic exposure to paclitaxel (Figure 4B) were not dependent on the administered formulation. Under these conditions, we found that changes in the concentration time–profile (Figure 4C) and peak concentration of CDCA-24G (Figure 4D) were more pronounced after the administration of Taxol compared with Abraxane, and similar observations were made for GCDCA-S (Figure 4E,F). These findings imply that Cremophor EL contributes to the observed changes in CDCA-24G plasma levels after treatment with Taxol. Consistent with this supposition, we found that the administration of Cremophor EL (without paclitaxel) caused significant increases in the plasma levels of CDCA-24G in wild-type mice but not in Oatp1b2(−/−) mice, although the effects associated with Cremophor EL given alone were less pronounced than those observed after administration of Taxol (Figure 4G and Figure S4).

**Figure 5:**
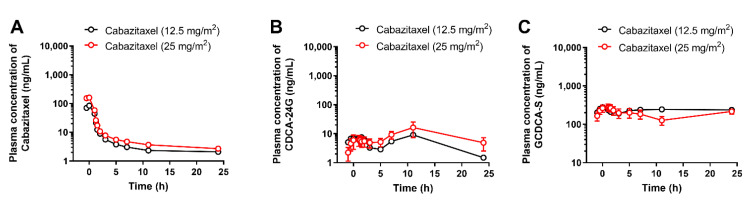
Influence of cabazitaxel on OATP1B biomarkers. Plasma concentrations of (**A**) cabazitaxel, (**B**) CDCA-24G, and (**C**) GCDCA-S in patients after i.v. infusion of cabazitaxel at doses of 12.5 or 25 mg/m^2^ (*n* = up to 10 per dose level). All data are presented as mean ± SEM.

For a biomarker to be considered clinically useful, the magnitude of its dependence on a particular transport mechanism should be sufficiently significant to allow for differentiation between weak, moderate, and strong inhibitors of the transporter in question. To address this issue for CDCA-24G, we next performed analyses on samples obtained from human subjects receiving the antineoplastic taxane cabazitaxel. Unlike paclitaxel [28], the degree to which OATP1B-type transporters contribute to the cellular uptake of cabazitaxel is controversial [28,29,30], and cabazitaxel is not anticipated to inhibit these transporters at concentrations associated with the approved dose of 25 mg/m^2^ [31]. In line with this prior information, we found that the administration of cabazitaxel (up to 25 mg/m^2^) to patients was not associated with elevated plasma levels of CDCA-24G and GCDCA-S (Figure 5).

## 4. Discussion

In the present study, we employed an untargeted metabolomics approach to transporter biomarker identification using various engineered mouse models and identified the conjugated bile acid CDCA-24G as a highly sensitive endogenous marker for hepatic OATP1B-type transporters. In replication studies, we found that the plasma levels of CDCA-24G at baseline were dramatically elevated in Oatp1b2(−/−) mice and that these levels increased acutely upon a challenge with the OATP1B inhibitors rifampin or paclitaxel in both wild-type mice and humans. The current study adds to a growing body of knowledge that SLCs belonging to the OATP1B family can have a dramatic impact on the hepatocellular processing of endogenous metabolites in a manner that is highly sensitive to certain prescription drugs, and as such, the present findings provide an opportunity for a priori determination of an individual’s hepatic OATP1B activity and predict or prevent unwarranted DDIs in subjects receiving polypharmacy treatment regimens with OATP1B inhibitors and substrates.

Two of the most commonly acknowledged risk factors of potentially harmful DDIs are polypharmacy and advanced age [32,33,34]. In the field of oncology, for example, recent investigations have demonstrated that as many as 30% of cancer patients receiving chemotherapy are at a risk for DDIs [35]. As the number of new treatment options in virtually all therapeutic areas continues to grow, DDIs are increasingly recognized as significant health hazards that can negatively influence treatment outcomes. These issues are particularly concerning given the increasing use of polypharmacy and orally administered medications. While the latter offer advantages in terms of patient preference and convenience of use, it has been suggested that the use of such agents increases the risk of potentially serious DDIs with many widely used outpatient medications [36]. Indeed, while the prevalence of DDIs with oral drugs is as high as 50% with nearly 20% of these being associated with increased toxicities, the mechanistic basis, the clinical impact, and the contribution of OATP1B-type transporters to DDIs involving polypharmacy remain largely unknown and unstudied [33]. In the current study, we attempted to address this issue by exploring the utility of a bottom–up strategy to develop an endogenous biomarker for OATP1B-mediated transport of xenobiotics based on the assumption that this is an important contributing source of inter-individual variability in the toxic response to polypharmacy-based therapy.

The OATP1B biomarker identified in this study, CDCA-24G, belongs to the class of bile acids that has an important physiological role in emulsifying dietary fats, eliminating cholesterol, and clearing hepatic catabolites. Bile acids are typically synthesized in the liver by cytochrome P450-mediated oxidation of cholesterol and are stored in the gall bladder and released into the small intestine [37]. The primary bile acids cholate and chenodeoxycholate are conjugated, secreted into the bile [38,39], and can undergo deconjugation by bacterial enzymes present in the gut to release the original secondary bile acids [37]. Via enterohepatic circulation, these compounds are then transported back to the liver and recycled. Multiple bile acids were previously reported to be transported substrates of OATP1B [21,22], and this prior knowledge is consistent with the observation from our untargeted metabolomics study that numerous primary and secondary bile acids were significantly deregulated in Oatp1b2(−/−) mice. No apparent differences in ex vivo glucuronide formation rates were previously observed between Oatp1b2(−/−) mice and wild-type mice [24], suggesting that the observed phenotypic differences were due to aberrant hepatic uptake. One possible limitation of the Oatp1b2(−/−) model is the notion that, unlike in humans, mouse hepatocytes express multiple members of Oatp1a, which is a related subfamily of transporters that can potentially provide compensatory restoration of function when Oatp1b2 is lost [25]. However, compared to the corresponding wild-type mice, increases in the plasma concentrations of CDCA-24G were also observed in Oatp1a/1b(−/−) mice, which are additionally deficient in all Oatp1a isoforms. Similar phenotypic changes have previously been reported to occur for the glururonide metabolites of the kinase inhibitors sorafenib [24,40] and regorafenib [41] as well as for conjugated bilirubin [42], whereby the deficiency of either Oatp1b2 or both Oatp1a and Oatp1b2 leads to an excessive buildup of glucuronides in the systemic circulation. It is worth noting that some recent identified potential biomarkers in humans including glycodeoxycholate 3-O-glucuronide [43] were not identified from our metabolomics study; this discrepancy could point to species differences, as reported in another study [44]. CDCA-24G, a bile acid with high relative abundance in both human and rodent [19,20], was further considered for prospective validation, since our intention was to derive species-independent biomarkers of hepatic OATP1B function. Of the four biomarkers we have quantified, CDCA-24G was identified with the highest relative abundance in mice plasma versus GCDCA-S in human plasma at baseline with the same sensitivity of analytical method for both analytes. Previous studies also suggested that the inter-individual and inter-day baseline variation of the plasma CDCA-24G is very minor compared to other endogenous biomarkers [13,20,45].

In our current study, the introduction of human OATP1B1 or human OATP1B3 into the liver of Oatp1a/1b(−/−) mice resulted only in partially restored function to the baseline levels of CDCA-24G observed in wild-type mice. It is possible that this lack of complete functional restoration is due to differences in affinity of CDCA-24G for Oatp1b2 relative to OATP1B1 and OATP1B3, which is a conclusion that is consistent with our present in vitro data. In addition, it cannot be excluded that the hepatic expression of OATP1B1 or OATP1B3 in the transgenic mice is lower than that of Oatp1b2 in the parental strains or that full functional restoration could have been achieved in Oatp1a/1b(−/−) mice expressing both OATP1B1 and OATP1B3. Regardless of the underlying mechanism, these data support the possibility that even partial changes in the collective function of OATP1B1 and OATP1B3, for example because of an inherited genetic defect in one or both of these transporters, can lead to a buildup of CDCA-24G in the circulation and result in an altered recycling of bile acids. This possibility is consistent with the notion that the processing of certain glucuronidated bile acids is severely compromised in subjects with Rotor syndrome, which is a condition associated with the complete inactivation of OATP1B1 and OATP1B3 [46].

Beyond CDCA-24G, a multitude of additional endogenous substrates of OATP1B have been identified during the past decade, and some of these compounds have been proposed as potential biomarkers in conjunction with clinical evaluations of OATP1B-mediated DDIs [3,4,47]. For example, plasma levels of biomarkers such as direct bilirubin, GCDCA-S, and hexadecanedioate are sensitive to differing degrees to treatment with the OATP1B inhibitor rifampin in healthy volunteers in a manner that is dependent on the rifampin dose [45]. In addition, the coproporphyrins CP-I and CP-III and certain bile acids have been applied as endogenous OATP1B biomarkers to confirm the in vivo inhibitory potency of paclitaxel in patients with non-small cell lung cancer [20]. These observations are consistent with our current finding that plasma levels of CDCA-24G are elevated following the administration of paclitaxel at therapeutic doses in both mice and human patients. In contrast, administration of the related anticancer drug cabazitaxel was not associated with altered levels of CDCA-24G, and this finding is consistent with the predicted limited influence of cabazitaxel on OATP1B-type transporters at clinically relevant doses [31]. The main intention of our collective studies is to validate biomarkers identified from the metabolomics study in mice, supporting that the bottom–up methodology is a valuable, understudied alternative to more commonly applied top–down approaches. These studies do not invalidate the potential utility of previously proposed OATP1B biomarkers in the context of evaluating DDI liabilities, and also, we are not claiming that CDCA-24G offers improvements in sensitivity as a biomarker.

We previously reported that the presence of Cremophor EL, the pharmaceutical vehicle used to solubilize paclitaxel in certain clinical preparations, even in relatively low amounts, can nullify the transport of OATP1B substrates [48,49]. Based on our current in vivo studies, it appears that the influence of paclitaxel-based treatment on OATP1B activity, derived from changes in the levels of CDCA-24G, is strongly enhanced in the presence of Cremophor EL. The notion that a relatively inert excipient such as Cremophor EL can directly inhibit OATP1B-type transporters may have important ramifications for the proper interpretation and safety of combination regimens that include paclitaxel or other agents formulated with this excipient (Table S2). For example, it can be postulated that intrinsic physiologic and environmental variables influencing OATP1B1- or OATP1B3-mediated uptake of paclitaxel itself into hepatocytes may have a more profound influence on drug clearance for formulations lacking solubilizers such as Cremophor EL. Since substrates of OATP1B1 and OATP1B3 for which the liver is the main organ of elimination are highly liable to DDIs associated with these transporters [50], our present findings strongly suggest that such interactions at the level of hepatocellular uptake mechanisms would be attenuated with Cremophor EL-free formulation of paclitaxel such as Abraxane. Conversely, Cremophor EL may act as a perpetrator in known DDIs involving other OATP1B substrates co-administered with paclitaxel (Taxol) [51], such as etoposide [52], docetaxel [53], oxaliplatin [54], or the irinotecan metabolite, SN-38 [55]. The mechanistic confirmation of this hypothesis is currently ongoing.

## 5. Conclusions

In conclusion, our data supported CDCA-24G as a highly sensitive and reliable endogenous biomarker of OATP1B-type transporters from a bottom–up metabolomics screening strategy employing various preclinical model organisms and human subjects treated with taxane antineoplastic drugs. In addition, our findings emphasize the importance of considering formulation excipients as perpetrators of OATP1B-dependent DDIs, shed light on the feasibility of transporter mediated DDI risk assessment in clinical settings, and provide a useful methodology for the future development of endogenous biomarkers for other important xenobiotic transporters.

## Data Availability

The data presented in this study are available on request from the corresponding author.

## Supplementary Materials

The following supporting information can be downloaded at: https://www.mdpi.com/article/10.3390/pharmaceutics14091933/s1, Figure S1: Global untargeted metabolomics study from plasma of wild-type mice or Oatp1b2-deficient mice. (A) Principal component analysis (PCA) score plots based on the metabolic profiles. (B) Pathway analysis of primary bile acid metabolism and secondary bile acid metabolism; Figure S2: Paclitaxel mediated inhibition of (A) CDCA-24G, (B) GCDCA-S, (C) CP-I, and (D) CP-III uptake in HEK293 cells overexpressing OATP1B1 or Oatp1b2. Uptake is expressed as percentage change compared with empty vector controls (*n* = 3 per group). All data are presented as mean ± SEM; Figure S3: Plasma levels of (A) CP-I, and (B) CP-III in wild-type mice, Oatp1a/1b(−/−) mice, OATP1B1(tg) mice, and OATP1B3 (tg) mice (*n* = 6–10 per group). Plasma concentration–time profiles of CDCA-24G in wild-type mice receiving (C) rifampin (20 mg/kg, i.v.) or (D) paclitaxel (10 mg/kg, i.v.), All data are presented as mean ± SEM; Figure S4: (A) Plasma concentration–time profiles of paclitaxel in wild-type mice and Oatp1b2-deficient mice. (B) Plasma concentration–time profiles of CDCA-24G in Oatp1b2-deficient mice, and (C) C_max_ fold changes of CDCA-24G in wild-type mice and Oatp1b2-deficient mice receiving Cremophor EL/ethanol (1:1, *v*/*v*, 5 mL/kg, i.v.) or paclitaxel (10 mg/kg, 5 mL/kg, i.v.), (*n* = 3–5 per group). All data are presented as mean ± SEM; Table S1: Differentially detected endogenous metabolites in untreated plasma of wild-type (WT) mice or Oatp1b2-deficient (KO) mice. Positive fold change indicates higher plasma concentration in Oatp1b2-deficient mice. Statistical analysis was performed using Welch’s two-sample *t*-test. Table S2: The inhibition of OATP1B1 and OATP1B3 transporters.
