# Supplementary material for: A Metabolomics Approach for Predicting OATP1B-Type Transporter-Mediated Drug–Drug Interaction Liabilities
Source: Pharmaceutics. 2022 Sep 13;14(9):1933. doi: 10.3390/pharmaceutics14091933 (PMC9502272; doi:10.3390/pharmaceutics14091933)
Supplement: Supplementary file 1 [file pharmaceutics-14-01933-s001.zip › pharmaceutics-1878447-SI.pdf]

Supplementary information for

A metabolomics approach for predicting OATP1B-type transporter-mediated drug-drug interaction liabilities

Yang Li[1,2], Yan Jin[1], Hanieh Taheri[1,2], Keith T. Schmidt[3], Alice A. Gibson[1], Stefan A. J. Buck[4], Eric D. Eisenmann[1], Ron H. J. Mathijssen[4], William D. Figg[3], Sharyn D. Baker[1], Alex Sparreboom[1], and Shuiying Hu[1,2]*

[1] Division of Pharmaceutics and Pharmacology, College of Pharmacy & Comprehensive Cancer Center, The Ohio State University, Columbus, Ohio 43210, USA

[2] Division of Outcomes and Translational Sciences, College of Pharmacy & Comprehensive Cancer Center, The Ohio State University, Columbus, Ohio 43210, USA

[3] Clinical Pharmacology Program, Office of the Clinical Director, National Cancer Institute, Bethesda, Maryland 20892, USA

[4] Department of Medical Oncology, Erasmus Medical Center Cancer Institute, Dr. Molewaterplein 40, 3015 GD Rotterdam, The Netherlands

* Correspondence: hu.1333@osu.edu; Tel.: +1-614-685-8028

Supplementary Information Files:

1. Figure S1

2. Figure S2

3. Figure S3

4. Figure S4

5. Table S1

6. Table S2

Figure S1

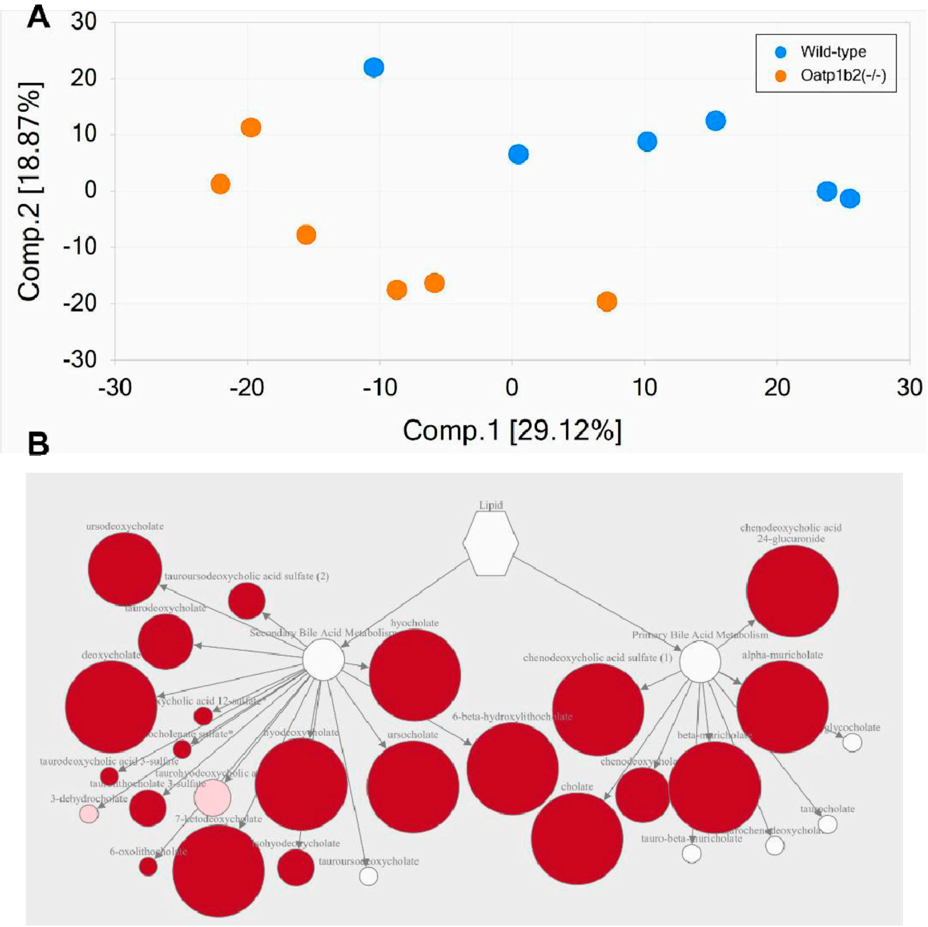

Figure S1 Global untargeted metabolomics study from plasma of wild-type mice or Oatp1b2-deficient mice. (A) Principal component analysis (PCA) score plots based on the metabolic profiles. (B) Pathway analysis of primary bile acid metabolism and secondary bile acid metabolism.

Figure S2

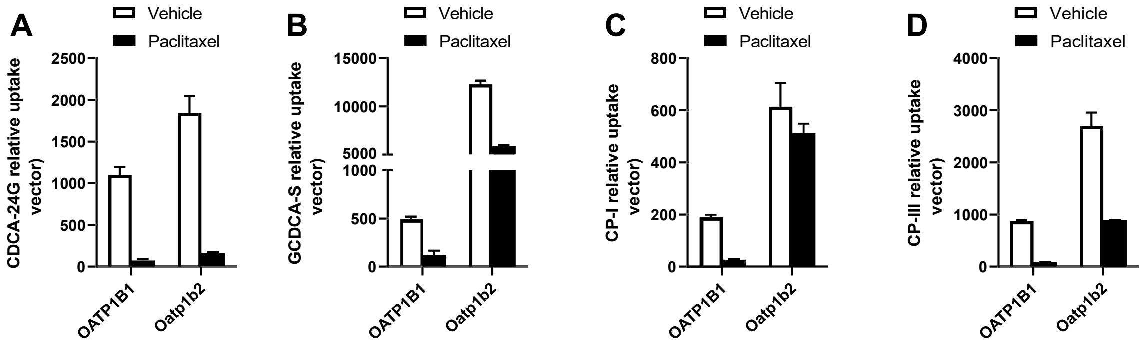

Figure S2 Paclitaxel mediated inhibition of (A) CDCA-24G, (B) GCDCA-S, (C) CP-I, and (D) CP-III uptake in HEK293 cells overexpressing OATP1B1 or Oatp1b2. Uptake is expressed as percentage change compared with empty vector controls (*n* = 3 per group). All data are presented as mean ± SEM.

Figure S3

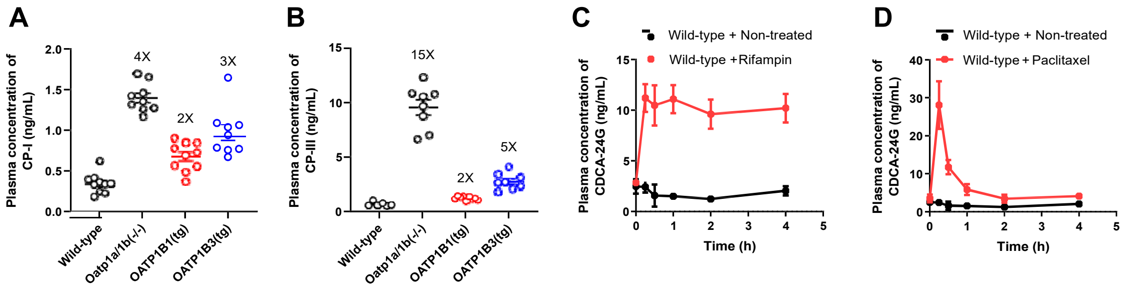

Figure S3 Plasma levels of (A) CP-I, and (B) CP-III in wild-type mice, Oatp1a/1b(-/-) mice, OATP1B1(tg) mice, and OATP1B3 (tg) mice (*n* = 6-10 per group). Plasma concentration-time profiles of CDCA-24G in wild-type mice receiving (C) rifampin (20 mg/kg, i.v.) or (D) paclitaxel (10 mg/kg, i.v.), All data are presented as mean ± SEM.

Figure S4

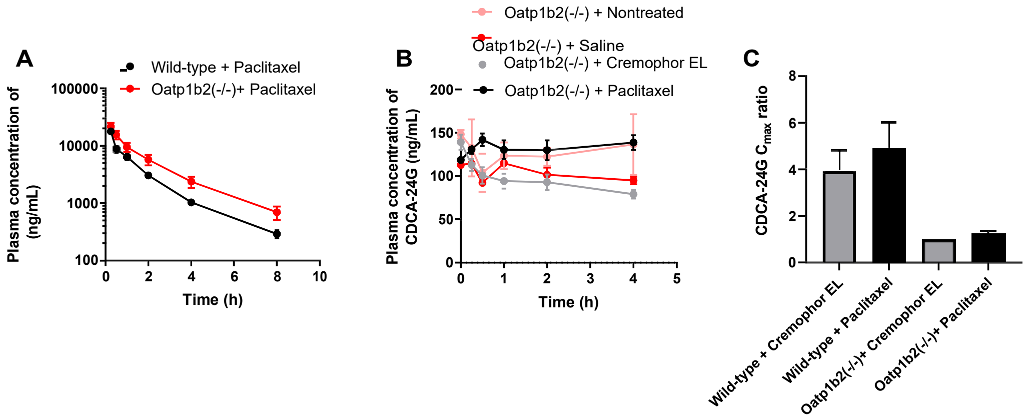

Figure S4 (A) Plasma concentration time profiles of paclitaxel in wild-type mice and Oatp1b2-deficient mice. (B) Plasma concentration time profiles of CDCA-24G in Oatp1b2-deficient mice, and (C) C_{max} fold changes of CDCA-24G in wild-type mice and Oatp1b2-deficient mice receiving Cremophor EL/ethanol (1:1, v/v, 5 mL/kg, i.v.) or paclitaxel (10 mg/kg, 5 mL/kg, i.v.), (*n* = 3-5 per group). All data are presented as mean ± SEM.

Table S1. Differentially detected endogenous metabolites in untreated plasma of wild-type (WT) mice or Oatp1b2-deficient (KO) mice. Positive fold change indicates higher plasma concentration in Oatp1b2-deficient mice. Statistical analysis was performed using Welch's two-sample *t*-test.

Biochemical Name	Fold of Change KO/WT	*P*-value KO/WT	Mean value KO	Mean value WT	Sub Pathway
chenodeoxycholic acid 24-glucuronide	97.9	<0.001	1.15	.0120	Primary Bile Acid Metabolism
alpha-muricholate	45.2	<0.001	2.71	.0600	Primary Bile Acid Metabolism
ursocholate	33.8	<0.001	5.10	.151	Secondary Bile Acid Metabolism
4-ethylphenylsulfate	17.2	<0.001	4.08	.238	Benzoate Metabolism
beta-muricholate	15.2	<0.001	2.84	.187	Primary Bile Acid Metabolism
hyodeoxycholate	14.7	<0.001	3.94	.269	Secondary Bile Acid Metabolism
cholate	13.7	<0.001	3.61	.263	Primary Bile Acid Metabolism
6-beta-hydroxylithocholate	13.1	<0.001	1.16	.0890	Secondary Bile Acid Metabolism
octadecenedioylcarnitine (C18:1-DC)*	12.7	<0.001	2.35	.185	Fatty Acid Metabolism (Acyl Carnitine, Dicarboxylate)
p-cresol glucuronide*	11.8	<0.001	5.92	.503	Tyrosine Metabolism
deoxycholate	10.0	<0.001	2.50	.249	Secondary Bile Acid Metabolism
p-cresol sulfate	9.29	<0.001	4.66	.501	Benzoate Metabolism
1-oleoyl-GPG (18:1)*	8.19	<0.001	1.80	.219	Lysophospholipid
dodecanedioate (C12-DC)	8.12	<0.001	2.97	.366	Fatty Acid, Dicarboxylate
bilirubin degradation product, C17H20N2O5 (1)**	7.14	<0.001	2.40	.336	Partially Characterized Molecules
bilirubin degradation product, C16H18N2O5 (1)**	6.67	<0.001	1.23	.185	Partially Characterized Molecules
branched chain 14:0 dicarboxylic acid**	6.55	<0.001	1.80	.275	Fatty Acid, Dicarboxylate
chenodeoxycholic acid sulfate (1)	6.41	0.006	1.02	.160	Primary Bile Acid Metabolism
7-ketodeoxycholate	6.23	0.001	2.26	.362	Secondary Bile Acid Metabolism
bilirubin degradation product, C16H18N2O5 (3)**	6.13	<0.001	1.96	.319	Partially Characterized Molecules
bilirubin (E,E)*	6.12	<0.001	1.17	.191	Hemoglobin and Porphyrin Metabolism
hyocholate	5.64	<0.001	1.41	.251	Secondary Bile Acid Metabolism
bilirubin degradation product, C17H18N2O4 (2)**	5.59	<0.001	2.14	.382	Partially Characterized Molecules
1-palmitoyl-GPG (16:0)*	5.40	<0.001	2.00	.371	Lysophospholipid
eicosenedioate (C20:1-DC)*	5.36	<0.001	1.80	.335	Fatty Acid, Dicarboxylate
bilirubin (Z,Z)	5.25	0.001	1.48	.282	Hemoglobin and Porphyrin Metabolism
bilirubin degradation product, C17H18N2O4 (1)**	5.05	<0.001	2.10	.416	Partially Characterized Molecules
3-hydroxydodecanedioate*	4.32	<0.001	1.64	.380	Fatty Acid, Dicarboxylate
2-amino-p-cresol sulfate	4.28	0.001	1.86	.435	Benzoate Metabolism
tetradecanedioate (C14-DC)	4.27	<0.001	1.96	.459	Fatty Acid, Dicarboxylate
1-stearoyl-GPG (18:0)	4.16	<0.001	1.76	.423	Lysophospholipid
biliverdin	4.09	<0.001	1.84	.450	Hemoglobin and Porphyrin Metabolism

ursodeoxycholate	4.07	0.007	2.16	.530	Secondary Bile Acid Metabolism
dodecenedioate (C12:1-DC)*	3.94	<0.001	2.14	.543	Fatty Acid, Dicarboxylate
1-linoleoyl-GPG (18:2)*	3.71	<0.001	1.96	.529	Lysophospholipid
bilirubin degradation product, C17H18N2O4 (3)**	3.65	<0.001	1.81	.496	Partially Characterized Molecules
bilirubin degradation product, C16H18N2O5 (2)**	3.62	<0.001	1.75	.482	Partially Characterized Molecules
hexadecenedioate (C16:1-DC)*	3.61	<0.001	1.62	.448	Fatty Acid, Dicarboxylate
chenodeoxycholate	3.57	<0.001	2.09	.585	Primary Bile Acid Metabolism
bilirubin degradation product, C17H20N2O5 (2)**	3.53	<0.001	2.00	.567	Partially Characterized Molecules
octadecadienedioate (C18:2-DC)*	3.48	<0.001	1.88	.540	Fatty Acid, Dicarboxylate
N,N,N-trimethyl-5-aminovalerate	3.45	<0.001	2.06	.597	Lysine Metabolism
taurodeoxycholate	3.45	0.001	1.82	.528	Secondary Bile Acid Metabolism
enterolactone sulfate	3.42	<0.001	1.63	.478	Food Component/Plant
glucuronate	3.17	<0.001	1.59	.500	Aminosugar Metabolism
2-hydroxy-3-methylvalerate	3.15	0.004	2.33	.738	Leucine, Isoleucine and Valine Metabolism
bilirubin degradation product, C16H18N2O5 (4)**	3.06	<0.001	1.58	.514	Partially Characterized Molecules
undecanedioate (C11-DC)	3.00	<0.001	1.96	.654	Fatty Acid, Dicarboxylate
octadecenedioate (C18:1-DC)	2.66	<0.001	1.45	.544	Fatty Acid, Dicarboxylate
equol sulfate	2.58	<0.001	1.62	.626	Food Component/Plant
isohyodeoxycholate	2.46	0.009	0.940	.382	Secondary Bile Acid Metabolism
phenylpropionylglycine	2.41	0.006	1.58	.656	Benzoate Metabolism
1-linoleoyl-GPA (18:2)*	2.36	0.003	1.41	.595	Lysophospholipid
alpha-hydroxyisocaproate	2.29	0.01	1.80	.787	Leucine, Isoleucine and Valine Metabolism
taurolithocholate 3-sulfate	2.25	0.02	0.960	.425	Secondary Bile Acid Metabolism
tauroursodeoxycholic acid sulfate (2)	2.18	0.01	1.12	.513	Secondary Bile Acid Metabolism
1-(1-enyl-oleoyl)-GPE (P-18:1)*	2.16	<0.001	1.45	.672	Lysoplasmalogen
pregnenediol disulfate (C21H34O8S2)*	2.14	0.03	1.16	.540	Pregnenolone Steroids
4-acetamidobenzoate	2.14	0.004	1.46	.685	Chemical
1-linolenoyl-GPC (18:3)*	2.13	<0.001	1.41	.661	Lysophospholipid
glucuronide of C10H18O2 (11)*	2.12	0.001	1.57	.737	Partially Characterized Molecules
glucuronide of C14H22O4 (1)*	2.12	<0.001	1.47	.690	Partially Characterized Molecules
hexadecanedioate (C16-DC)	2.10	0.001	1.57	.749	Fatty Acid, Dicarboxylate
tetradecadienedioate (C14:2-DC)*	2.09	0.006	1.58	.756	Fatty Acid, Dicarboxylate
inosine	2.07	0.03	1.49	.719	Purine Metabolism, (Hypo)Xanthine/Inosine containing
1-oleoyl-GPE (18:1)	2.05	<0.001	1.30	.634	Lysophospholipid
α-tocopherol	2.03	<0.001	1.44	.707	Tocopherol Metabolism
1,2-dipalmitoyl-GPE (16:0/16:0)*	2.02	0.001	1.34	.664	Phosphatidylethanolamine (PE)
1-oleoyl-2-arachidonoyl-GPI (18:1/20:4)*	2.01	0.01	1.37	.681	Phosphatidylinositol (PI)
cinnamoylglycine	1.99	0.02	1.54	.776	Food Component/Plant

1-palmitoleoyl-GPC (16:1)*	1.97	<0.001	1.31	.666	Lysophospholipid
2-palmitoleoyl-GPC (16:1)*	1.95	0.002	1.36	.696	Lysophospholipid
1-linoleoyl-GPE (18:2)*	1.94	0.004	1.42	.736	Lysophospholipid
4-ethylcatechol sulfate	1.93	0.001	1.56	.807	Benzoate Metabolism
arachidonoylcarnitine (C20:4)	1.92	<0.001	1.53	.796	Fatty Acid Metabolism (Acyl Carnitine, Polyunsaturated)
linolenoylcarnitine (C18:3)*	1.91	<0.001	1.51	.794	Fatty Acid Metabolism (Acyl Carnitine, Polyunsaturated)
equol glucuronide	1.90	0.006	1.35	.709	Food Component/Plant
phenol glucuronide	1.87	0.02	1.28	.684	Tyrosine Metabolism
1-oleoyl-GPI (18:1)	1.87	0.002	1.36	.729	Lysophospholipid
1-palmitoleoyl-2-linolenoyl-GPC (16:1/18:3)*	1.86	0.002	1.46	.784	Phosphatidylcholine (PC)
5-methyl-2'-deoxycytidine	1.83	0.002	1.56	.852	Pyrimidine Metabolism, Cytidine containing
4-hydroxycinnamate sulfate	1.81	0.009	1.28	.707	Tyrosine Metabolism
1-arachidonoyl-GPA (20:4)	1.80	0.008	1.17	.652	Lysophospholipid
1-(1-enyl-palmitoyl)-GPE (P-16:0)*	1.79	<0.001	1.30	.725	Lysoplasmalogen
1-oleoyl-GPC (18:1)	1.78	<0.001	1.30	.732	Lysophospholipid
2,8-quinolinediol sulfate	1.78	0.03	1.68	.946	Food Component/Plant
pentadecanoylcarnitine (C15)*	1.77	<0.001	1.28	.723	Fatty Acid Metabolism (Acyl Carnitine, Long Chain Saturated)
pyridoxamine	1.77	<0.001	1.42	.805	Vitamin B6 Metabolism
dihomo-linoleoylcarnitine (C20:2)*	1.75	<0.001	1.37	.785	Fatty Acid Metabolism (Acyl Carnitine, Polyunsaturated)
1-palmitoyl-GPE (16:0)	1.73	<0.001	1.33	.765	Lysophospholipid
butyrylglycine	1.72	0.007	1.28	.744	Fatty Acid Metabolism (also BCAA Metabolism)
dihomo-linolenoylcarnitine (C20:3n3 or 6)*	1.72	<0.001	1.37	.795	Fatty Acid Metabolism (Acyl Carnitine, Polyunsaturated)
1-(1-enyl-stearoyl)-GPE (P-18:0)*	1.72	<0.001	1.28	.747	Lysoplasmalogen
alpha-hydroxyisovalerate	1.68	0.003	1.43	.850	Leucine, Isoleucine and Valine Metabolism
heptadecanedioate (C17-DC)	1.66	0.001	1.36	.818	Fatty Acid, Dicarboxylate
1-linoleoyl-GPI (18:2)*	1.65	0.02	1.27	.765	Lysophospholipid
1-ribosyl-imidazoleacetate*	1.64	0.05	1.34	.818	Histidine Metabolism
palmitoleoylcarnitine (C16:1)*	1.63	0.02	1.54	.943	Fatty Acid Metabolism (Acyl Carnitine, Monounsaturated)
1-arachidonoyl-GPC (20:4n6)*	1.63	<0.001	1.27	.775	Lysophospholipid
octanoylcarnitine (C8)	1.62	0.03	1.47	.910	Fatty Acid Metabolism (Acyl Carnitine, Medium Chain)
linoleoylcarnitine (C18:2)*	1.62	0.004	1.36	.838	Fatty Acid Metabolism (Acyl Carnitine, Polyunsaturated)
1-linoleoyl-GPC (18:2)	1.62	<0.001	1.22	.752	Lysophospholipid
3-phenylpropionate (hydrocinnamate)	1.62	0.04	1.39	.855	Benzoate Metabolism
cinnamate	1.60	0.04	1.56	.975	Food Component/Plant
hexanoylglycine	1.59	0.05	1.40	.880	Fatty Acid Metabolism (Acyl Glycine)
margaroylcarnitine (C17)*	1.59	0.001	1.21	.764	Fatty Acid Metabolism (Acyl Carnitine, Long Chain Saturated)
1-(1-enyl-palmitoyl)-GPC (P-16:0)*	1.59	<0.001	1.26	.795	Lysoplasmalogen
taurodeoxycholic acid 3-sulfate	1.58	0.04	1.10	.694	Secondary Bile Acid Metabolism

isocaproylglycine	1.57	0.02	1.28	.815	Fatty Acid Metabolism (Acyl Glycine)
glycerophosphoethanolamine	1.57	0.002	1.31	.831	Phospholipid Metabolism
oleoylcarnitine (C18:1)	1.56	0.02	1.40	.900	Fatty Acid Metabolism (Acyl Carnitine, Monounsaturated)
mannitol/sorbitol	1.55	0.008	1.24	.800	Fructose, Mannose and Galactose Metabolism
4-vinylphenol sulfate	1.55	0.004	1.32	.847	Benzoate Metabolism
hydroquinone sulfate	1.55	0.02	1.32	.849	Drug - Topical Agents
valerylglycine	1.53	0.03	1.28	.838	Fatty Acid Metabolism (Acyl Glycine)
heptanoyl glycine	1.52	0.05	1.32	.868	Fatty Acid Metabolism (Acyl Glycine)
1-linoleoyl-2-linolenoyl-GPC (18:2/18:3)*	1.51	0.03	1.40	.923	Phosphatidylcholine (PC)
indolin-2-one	1.51	0.01	1.20	.797	Food Component/Plant
stearamide (18:0)	1.49	0.01	1.19	.800	Fatty Acid, Amide
1-arachidonoyl-GPE (20:4n6)*	1.49	0.01	1.20	.803	Lysophospholipid
6-hydroxyindole sulfate	1.49	0.01	1.14	.762	Chemical
3-indoxyl sulfate	1.48	0.02	1.19	.804	Tryptophan Metabolism
1-linoleoyl-2-arachidonoyl-GPE (18:2/20:4)*	1.48	0.03	1.26	.854	Phosphatidylethanolamine (PE)
1-arachidonoyl-GPI (20:4)*	1.48	0.02	1.11	.754	Lysophospholipid
pyridoxal	1.47	<0.001	1.28	.868	Vitamin B6 Metabolism
phenylacetylglutamate	1.46	0.04	1.24	.850	Acetylated Peptides
eicosadienamide (20:2)*	1.46	0.02	1.27	.869	Fatty Acid, Amide
5-dodecenoylcarnitine (C12:1)	1.46	0.04	1.37	.936	Fatty Acid Metabolism (Acyl Carnitine, Monounsaturated)
1-palmitoyl-2-oleoyl-GPE (16:0/18:1)	1.46	0.04	1.24	.847	Phosphatidylethanolamine (PE)
1-(1-enyl-palmitoyl)-2-arachidonoyl-GPC (P-16:0/20:4)*	1.46	0.001	1.30	.891	Plasmalogen
margaramide (17:0)*	1.45	0.009	1.19	.826	Fatty Acid, Amide
sebacate (C10-DC)	1.44	0.02	1.20	.833	Fatty Acid, Dicarboxylate
nonadecanedioate (C19-DC)	1.44	0.005	1.30	.902	Fatty Acid, Dicarboxylate
eicosenamide (20:1)*	1.43	0.03	1.27	.885	Fatty Acid, Amide
6-oxolithocholate	1.43	0.04	0.970	.678	Secondary Bile Acid Metabolism
N-acetylkynurenine (2)	1.41	0.003	1.17	.827	Tryptophan Metabolism
ethyl beta-glucopyranoside	1.41	0.02	1.17	.826	Food Component/Plant
phenol sulfate	1.40	0.03	1.23	.879	Tyrosine Metabolism
beta-hydroxyisovalerate	1.40	0.005	1.17	.835	Leucine, Isoleucine and Valine Metabolism
isobutyrylcarnitine (C4)	1.40	0.02	1.15	.817	Leucine, Isoleucine and Valine Metabolism
N-acetylmethionine sulfoxide	1.40	0.05	1.31	.931	Methionine, Cysteine, SAM and Taurine Metabolism
palmitamide (16:0)	1.40	0.007	1.18	.840	Fatty Acid, Amide
nonadecenamide (19:1)*	1.39	0.03	1.27	.917	Fatty Acid, Amide
myristoylcarnitine (C14)	1.39	0.04	1.26	.904	Fatty Acid Metabolism (Acyl Carnitine, Long Chain Saturated)
tigloylglycine	1.38	0.02	1.22	.883	Leucine, Isoleucine and Valine Metabolism
octadecanedioate (C18-DC)	1.38	0.003	1.27	.916	Fatty Acid, Dicarboxylate

oleamide	1.38	0.01	1.22	.886	Fatty Acid, Amide
heptadecenamide (17:1)*	1.37	0.03	1.29	.939	Fatty Acid, Amide
linoleamide (18:2n6)	1.36	0.04	1.29	.945	Fatty Acid, Amide
arachidoylcarnitine (C20)*	1.36	0.01	1.21	.889	Fatty Acid Metabolism (Acyl Carnitine, Long Chain Saturated)
1-linoleoyl-2-arachidonoyl-GPC (18:2/20:4n6)*	1.36	0.007	1.15	.846	Phosphatidylcholine (PC)
1-lignoceroyl-GPC (24:0)	1.36	0.007	1.16	.847	Lysophospholipid
sphingomyelin (d18:2/14:0, d18:1/14:1)*	1.36	0.03	1.14	.839	Sphingomyelins
N4-acetylcytidine	1.36	0.003	1.12	.819	Pyrimidine Metabolism, Cytidine containing
N-acetylhomocitrulline	1.35	0.001	1.17	.861	Urea cycle; Arginine and Proline Metabolism
2,3-dihydroxy-5-methylthio-4-pentenoate (DMTPA)*	1.34	0.01	1.23	.915	Methionine, Cysteine, SAM and Taurine Metabolism
glycerophosphorylcholine (GPC)	1.34	<0.001	1.12	.836	Phospholipid Metabolism
1-oleoyl-2-docosahexaenoyl-GPC (18:1/22:6)*	1.34	0.001	1.17	.874	Phosphatidylcholine (PC)
1-stearoyl-GPE (18:0)	1.34	0.002	1.16	.867	Lysophospholipid
1-(1-enyl-palmitoyl)-2-linoleoyl-GPE (P-16:0/18:2)*	1.34	0.03	1.16	.862	Plasmalogen
1-(1-enyl-palmitoyl)-2-arachidonoyl-GPE (P-16:0/20:4)*	1.34	0.005	1.13	.839	Plasmalogen
pyridoxate	1.34	0.02	1.21	.902	Vitamin B6 Metabolism
1-(1-enyl-stearoyl)-2-arachidonoyl-GPE (P-18:0/20:4)*	1.33	0.01	1.10	.828	Plasmalogen
1-(1-enyl-palmitoyl)-2-oleoyl-GPE (P-16:0/18:1)*	1.32	0.03	1.18	.895	Plasmalogen
sphingomyelin (d17:1/14:0, d16:1/15:0)*	1.32	0.03	1.02	.769	Sphingomyelins
1,2-dipalmitoyl-GPC (16:0/16:0)	1.31	0.02	1.18	.901	Phosphatidylcholine (PC)
erythritol	1.31	0.008	1.22	.927	Food Component/Plant
histidine betaine (hercynine)*	1.31	0.04	1.09	.830	Food Component/Plant
1-palmitoyl-2-oleoyl-GPC (16:0/18:1)	1.30	0.02	1.08	.834	Phosphatidylcholine (PC)
2-(4-hydroxyphenyl)propionate	1.30	0.01	1.16	.895	Benzoate Metabolism
1-palmitoyl-GPC (16:0)	1.29	<0.001	1.12	.868	Lysophospholipid
4-cholesten-3-one	1.29	0.01	1.12	.870	Sterol
taurocholenate sulfate*	1.29	0.05	1.08	.838	Secondary Bile Acid Metabolism
stachydrine	1.29	0.01	1.15	.893	Food Component/Plant
hypotaurine	1.28	0.009	1.14	.887	Methionine, Cysteine, SAM and Taurine Metabolism
7-alpha-hydroxy-3-oxo-4-cholestenoate (7-Hoca)	1.27	0.05	1.20	.944	Sterol
methylphosphate	1.27	0.02	1.08	.848	Purine and Pyrimidine Metabolism
3-hydroxy-2-ethylpropionate	1.25	0.01	1.10	.879	Leucine, Isoleucine and Valine Metabolism
deoxycholic acid 12-sulfate*	1.25	0.04	1.06	.851	Secondary Bile Acid Metabolism
pantothenate	1.25	0.01	1.07	.856	Pantothenate and CoA Metabolism
N-acetyl-3-methylhistidine*	1.24	0.006	1.09	.878	Histidine Metabolism
1-(1-enyl-palmitoyl)-2-palmitoyl-GPC (P-16:0/16:0)*	1.24	0.01	1.14	.919	Plasmalogen
mevalonate	1.24	0.05	1.15	.925	Mevalonate Metabolism
gamma-glutamylglycine	1.23	0.02	1.17	.950	Gamma-glutamyl Amino Acid

cholesterol sulfate	1.23	0.03	1.08	.875	Sterol
isocitric lactone	1.22	0.05	1.08	.884	TCA Cycle
1-stearoyl-2-oleoyl-GPC (18:0/18:1)	1.20	0.04	1.07	.892	Phosphatidylcholine (PC)
S-methylglutathione	1.19	0.04	1.11	.931	Glutathione Metabolism
homostachydrine*	1.19	0.04	1.09	.915	Food Component/Plant
octadecanedioylcarnitine (C18-DC)*	1.16	0.05	1.00	.860	Fatty Acid Metabolism (Acyl Carnitine, Dicarboxylate)
2-O-methylascorbic acid	1.16	0.03	1.08	.927	Ascorbate and Aldarate Metabolism
(N(1) + N(8))-acetylspermidine	1.15	0.02	1.07	.931	Polyamine Metabolism
1-palmitoyl-2-stearoyl-GPC (16:0/18:0)	1.14	0.01	1.09	.960	Phosphatidylcholine (PC)
sphingomyelin (d18:1/14:0, d16:1/16:0)*	1.14	0.04	1.06	.930	Sphingomyelins
sphingomyelin (d18:2/16:0, d18:1/16:1)*	1.13	0.02	1.06	.940	Sphingomyelins
pipecolate	1.12	0.03	1.05	.938	Lysine Metabolism
adenosine 5'-monophosphate (AMP)	1.12	0.04	1.04	.927	Purine Metabolism, Adenine containing
glycine	1.12	0.04	1.06	.944	Glycine, Serine and Threonine Metabolism

* Indicates compounds that have not been officially confirmed based on a standard, but we are confident in its identity.
** Indicates a compound for which a standard is not available, but we are reasonably confident in its identity or the information provided.

Table S2. The inhibition of OATP1B1 and OATP1B3 transporters.

Inhibitor	OATP1B1 IC_{50} (μM)	OATP1B3 IC_{50} (μM)	C_{max} (μM)	$f_{u,p}$ (%)	$1+((f_{u,p} \times C_{max})/IC_{50})$	Citation (PMID)
**Rifampin**	1.50	2.60	29.0	11.0	OATP1B1: 3.13<br>OATP1B3: 2.23	18321482<br>35456528
**Paclitaxel**	0.0300	0.500	9.60	3.60-7.90	OATP1B1: 26.3<br>OATP1B3: 2.52	18321482<br>28612269<br>10997930
**Cabazitaxel**	N/A	N/A	0.193	8.00-11.0	N/A	27796539<br>23299792
**Cremophor EL**	633	3.33	2490	100	OATP1B1: 4.92<br>OATP1B3: 746	11129729<br>22808947<br>12844327
